# Distinguishing Intergroup and Long-Distance Relationships

**DOI:** 10.1007/s12110-022-09431-1

**Published:** 2022-10-01

**Authors:** Anne C. Pisor, Cody T. Ross

**Affiliations:** 1 Department of Anthropology, Washington State University, Pullman, WA USA; 2 Department of Human Behavior, Ecology and Culture, Max Planck Institute for Evolutionary Anthropology, Leipzig, Germany

**Keywords:** Intergroup relations, Intergroup conflict, Cooperation, Sociality, Parochial altruism

## Abstract

Intergroup and long-distance relationships are both central features of human social life, but because intergroup relationships are emphasized in the literature, long-distance relationships are often overlooked. Here, we make the case that intergroup and long-distance relationships should be studied as distinct, albeit related, features of human sociality. First, we review the functions of both kinds of relationship: while both can be conduits for difficult-to-access resources, intergroup relationships can reduce intergroup conflict whereas long-distance relationships are especially effective at buffering widespread resource shortfalls. Second, to illustrate the importance of distinguishing the two relationship types, we present a case study from rural Bolivia. Combining ethnography and two different experimental techniques, we find that the importance of intergroup relationships—and the salience of group membership itself—varies across populations and across methods. Although ethnography revealed that participants often rely on long-distance relationships for resource access, we were unable to capture participant preferences for these relationships with a forced-choice technique. Taken together, our review and empirical data highlight that (1) intergroup and long-distance relationships can have different functions and can be more or less important in different contexts and (2) validating experimental field data with ethnography is crucial for work on human sociality. We close by outlining future directions for research on long-distance relationships in humans.

**Supplementary Information:**

The online version contains supplementary material available at 10.1007/s12110-022-09431-1.

Environmental variability is a quintessential feature of the human niche, and social connections spanning distance have long helped humans respond to variability (Pisor & Jones, 2021; Pisor & Surbeck, 2019; Sterelny, 2011). That said, the social science literature has focused on the study of symbolically marked groups, meaning that the importance of long-distance relationships is often overlooked—especially since many long-distance relationships also cross group boundaries (Pisor & Jones, 2021; Pisor & Surbeck, 2019). Here, we make the case that long-distance relationships and intergroup relationships should be studied as distinct, albeit related, features of human sociality.

We begin by reviewing the functions of intergroup and long-distance relationships and discussing the possible history of selection that may have favored each. Groups can serve as containers for cooperation in humans (Boyd & Richerson, 1985), directing favoritism toward in-group individuals and competition toward outsiders (Bowles & Choi, 2001; Bowles & Gintis, 2004). Intergroup relationships can reduce intergroup conflict (Brewer, 2010; Sherif, 1966) and provide other benefits, such as facilitating trade (Jha, 2013) and fostering resource specialization (Barth, 1998). However, specialization is not always organized by group; when resource availability differs across space—due to specialization, for example, or because shortfalls in resource availability are spatially correlated—intergroup relationships may or may not provide access (Pisor & Jones, 2021; Pisor & Surbeck, 2019). Instead, long-distance relationships can be key.

Given that intergroup and long-distance relationships can serve different functions, we draw on field data to assess the relevance of group membership and location in the choice of new social partners. In a multiyear project in rural Bolivia, author ACP used a variety of quantitative and qualitative methods to study intergroup and long-distance relationships with three populations of horticulturalists. She designed an economic game to examine whether individuals weigh group membership when choosing a new social partner (*n* = 200); in 2014–2015 she found that the salience of group membership varied across populations, reflecting variation in the frequency and valence of intergroup interactions. However, ethnographic data suggested that long-distance relationships were a central feature of rural Bolivian life. ACP came to speculate that distance might be more salient to partner choice in rural Bolivia than group membership per se.

To explicitly contrast preferences for forming intergroup versus long-distance relationships, ACP conducted a second study in 2017 among two of the three horticultural populations (*n* = 125) using a specially designed forced-choice task. She found that even though many participants actively maintain long-distance relationships, they did not express preferences for these relationships in the forced-choice task. This study underscored that (1) group membership and location are just two of many criteria used in determining the value of potential cooperative partners and (2) due to response biases, demand characteristics, and a disconnect from real-world incentives and constraints, the preferences elicited by experimental methods may not correlate with real-world behavior. Taken together, the results from Bolivia indicate that intergroup relations, both positive and negative, can be overemphasized, and long-distance relationships overlooked, if researchers studying human sociality do not differentiate between the two and do not cross-validate their results using multiple methods.

We conclude by discussing ideas for how researchers can better study long-distance relationships and review what we think should be the central foci of a research program characterizing the role of long-distance relationships in human sociality.

## Distinguishing Intergroup from Long-Distance Relationships

Intergroup relationships have long been a focus of social science research. In social psychology, *intergroup relations* is a commonly recognized subfield, whereas in evolutionary anthropology and evolutionary psychology, research on human sociality often focuses on *parochial altruism*—in-group favoritism coupled with out-group competition (e.g., Böhm et al., 2020; Pisor & Ross, 2022). Partially because of the extensive literatures on intergroup relations and parochial altruism, researchers are often primed to focus on groups when studying human sociality. Groups do structure human social networks, but as we detail here, long-distance relationships—which often cross group boundaries—are a central feature of these networks too (Jones et al., 2021).

Long-distance relationships are, of course, an implicit focus of fields studying trade, migration, intermarriage, and interactions with strangers—e.g., in archaeology (e.g., Minnis, 1985), geography (e.g., Newman & Dale, 2007), and psychology (e.g., Berry, 2001). Each is important to human responses to environmental variability, and thus to human adaptation (Pisor & Jones, 2021; Pisor & Surbeck, 2019). That said, these different types of relationships (e.g., with trading partners, host families, and affinal kin) are usually not studied as part of a single category: relationships that typically span distance.

### What Are Intergroup Relationships?

Social scientists often use the word “group” without specifying what a group is (Pietraszewski, 2022). In social psychology, two individuals are usually considered to be part of the same group if they have a shared fate or a shared identity (Böhm et al., 2020); these groups are considered “natural” groups if they are not created in the laboratory as part of an experiment (Balliet et al., 2014). When evolutionary anthropologists or evolutionary psychologists refer to groups, they too are generally referring to identity groups, especially when identity is observable to others (Smaldino, 2019), when identity is symbolically marked (Moya & Boyd, 2015), or when identity is assigned to individuals by virtue of their relationships with third parties (Pietraszewski, 2022). Alternatively, consistent with the literature on animal behavior, evolutionary scholars may refer to groups as aggregations of individuals that are in physical proximity with one another more often than they are with others (Kummer, 1971).

When researchers study “natural” groups, they often focus on symbolically marked groups—for example, ethnic groups, religious groups, or groups based on regional/national identity—in which individuals share norms of behavior and an origin story (e.g., Barth, 1998; Berry, 2001). This is especially true in the parochial altruism literature, which usually focuses on favoritism toward members of the same ethnic group or (less frequently) members of the same religious group (Lang et al., 2019; Pisor & Ross, 2022). In keeping with common usage, we will generally refer to intergroup relationships as ties between two individuals that cross ethnic or religious boundaries.

Intergroup relationships are thought to have two key adaptive functions: they (1) facilitate resource specialization and (2) reduce costly intergroup conflict. For example, ethnic group identity is often structured around resource control or specialization (e.g., Cronk, 2004; Yount et al., 2001) and intergroup relationships can enable gains from trade across these boundaries (Barth, 1998; Jha, 2013). Intergroup relationships permit not only the flow of physical resources between groups but also the flow of ideas, norms, and technologies (Richerson et al., 2016). Additionally, intergroup relationships can reduce intergroup conflict (Pettigrew & Tropp, 2006). For example, intermarriage can be used to defuse tensions across group boundaries (Chapais, 2009).

### What Are Long-Distance Relationships?

Connections spanning distance are quite common across human societies, but their importance is often overlooked in the study of human sociality (Wobst, 1978). Individuals may form long-distance relationships when they engage in business, trade, or travel—and, today, such relationships can even be formed through the Internet. When the potential benefits of long-distance relationships are substantial, cultural institutions can emerge that reduce the costs of forming and maintaining long-distance relationships (Jha, 2013; Pisor & Surbeck, 2019). Such institutions may regulate the norms and expectations surrounding affinal relationships (Chapais, 2009), ritual relationships (e.g., stock friendships; Bollig, 2010), or patron-client relationships (Demps & Winterhalder, 2018).

There are two key benefits to long-distance relationships: risk management and nonlocal resource access (Pisor & Surbeck, 2019). Long-distance relationships allow individuals to manage the risk of resource shortfalls that can strike entire communities. Likewise, long-distance relationships can enable access to resources that are not locally available (tool-making materials, market goods, or even high-paying jobs) because they are clustered in space or because of specialization. Generally, as the distance between two individuals increases, shortfalls in resource access become less correlated and the diversity of resources the two can jointly access increases (Pisor & Jones, 2021). The distance required to achieve low correlation in shortfalls across a dyad will depend on the scale of the threats to resource security; for example, hurricanes can generate shortfalls impacting large geographic areas, whereas pests often impact smaller areas. Similarly, the distance required to achieve diversity in joint resource access depends on the local ecology—for example, how far one must travel to reach a different ecozone (Pisor & Jones, 2021). The high resource diversity made possible by long-distance relationships is particularly important to humans because of our reliance on rare, often difficult-to-obtain nutrients, minerals, and raw materials (Pisor & Surbeck, 2019).

Two quintessential ethnographic case studies provide examples of these key benefits—nonlocal resource access and risk management. San traditionally maintained *hxaro* relationships with both kin and putative kin living up to 200 km away (Wiessner, 1982). An individual’s hxaro partners often had access to resources that the individual’s band did not (such as health clinics and schools) but also to the same resources (such as game and water) which vary in their availability across time and space. When bands experienced correlated shortfalls—due, for example, to high winds or insect plagues—individuals would often leave to visit their hxaro partners for extended periods of time (Wiessner, 1977). In short, hxaro is a cultural institution that facilitates the formation and maintenance of long-distance relationships. Similarly, Massim traditionally practiced *kula*, a system of visitation and delayed exchange in which men would regularly visit their kula partners on different islands (Malinowski, 1922). Like hxaro, kula both buffered local shortfalls and provided nonlocal resource access: communities on some islands specialized in pottery, which other communities desired; when droughts struck, men from islands with limited arable land would visit partners on islands with larger food supplies (Irwin et al., 2019). Unlike hxaro, however, kula took place across linguistic boundaries (Irwin et al., 2019; Malinowski, 1922), underscoring that long-distance relationships *can* cross group boundaries, but need not.

Importantly, the resources that move between long-distance social connections are not limited to consumable or utilitarian resources. Kula, for example, provided access to prestige goods from other places (Irwin et al., 2019; Malinowski, 1922). Even in the absence of global communication systems or written language, cultural information—such as that contained in stories—can travel across large distances (Ross & Atkinson, 2016); in turn, this transmitted information is often pertinent to resource production (Boyd & Richerson, 1985), coordination (McConvell, 2015; Smith et al., 2017), and social organization (Yengoyan, 1968). Long-distance ties can be solidified by marriage—generating affinal relationships—and can also expand the marriage market (Yengoyan, 1968). Today, long-distance connections have become especially important for obtaining access to jobs (Bird et al., 2019; Pisor & Jones, 2021) or loans (Smith et al., 2022).

That said, long-distance relationships are not without their costs. In the absence of mass communication and transportation systems, maintenance of long-distance relationships can require visitation (Minnis, 1985)—costly, for example, due to lost production time and risk of attack during transit (Fitzhugh et al., 2011). Given such costs, we should only expect individuals to form, maintain, and desire long-distance relationships when the expected benefits of these relationships outweigh the expected costs (Minnis, 1985; Pisor & Jones, 2021).

### How Do Intergroup and Long-Distance Relationships Differ?

Intergroup relationships and long-distance relationships have partially overlapping functions, though they are by no means identical. Both intergroup relationships and long-distance relationships can offer access to resources that (1) are not accessible to in-group members or social partners living close by and/or (2) are less correlated with an individual’s own resource holdings than those held by in-group members or partners living close by. However, long-distance relationships do not necessarily cross group boundaries. Because shared norms and institutions can minimize the costs of within-group interactions (McElreath et al., 2003), the net benefits of long-distance relationships with in-group members will often be higher than the net benefits of long-distance relationships with out-group members (e.g., Ensminger, 1994; Purzycki et al., 2016). Long-distance relationships are also less likely to reduce parochial attitudes, which are best attenuated by frequent, positively valenced interactions (that is, interactions that go well) (Dovidio et al., 2003); such interactions are more likely when members of different groups live near one another (e.g., Bunce & McElreath, 2018). Indeed, because members of different groups often live close together, particularly when population density is high, intergroup relationships will not necessarily provide access to less-correlated resources—this risk-management function is actually more characteristic of long-distance relationships than intergroup relationships.

Evidence suggests that both intergroup and long-distance relationships have long been features of human sociality. Members of the genus *Homo* likely had ties spanning distance by at least 300,000 years ago (Foley & Gamble, 2009), if not 500,000 to 1 million years ago (Layton et al., 2012); periodic aggregation (such as seasonal meetings at productive resource patches; Kelly, 2007) might have reduced the cost of forming and maintaining these relationships. By the late Pleistocene, long-distance relationships appear to have been a central feature of human sociality (Foley & Gamble, 2009; Singh & Glowacki, 2022; Sterelny, 2011). The movement of durable goods—such as stone, shells, and other materials that persist in the archaeological record—across tens to hundreds of kilometers provides supporting evidence; though these goods may have passed through multiple hands, researchers point to the movement of goods as evidence for social networks spanning large distances (Foley & Gamble, 2009; Gamble, 1999; Irwin et al., 2019; Whallon, 2006). If this interpretation of the archaeological record is correct, long-distance relationships may have become a central feature of human sociality earlier than intergroup relationships.

Turning to intergroup relationships, resources that are monopolizable—either because they are clumped in space (e.g., Dyson-Hudson & Smith, 1978) or because groups can specialize in their production (e.g., Barth, 1956)—are likely to be defended. When resources are both valuable and defensible, institutions promoting the acquisition and defense of these resources are likely to emerge in human populations (Barth, 1998; Glowacki, 2020), often leading to sedentism (Kelly, 2007). Sedentism, in turn, increases the relevance of using identity markers to pick social partners since it becomes difficult to keep track of individualized characteristics as population size grows (Smaldino, 2019). Archaeologists often interpret stylistic decoration, such as that on pottery or beadwork, as indicative of identity markers. In the archaeological record, movement of symbolically marked goods across space may thus reflect intergroup relationships (e.g., Braun & Plog, 1982; Irwin et al., 2019; Wiessner, 1984) but may also indicate pillaging or the expansion of identity groups—for example, through conquest or colonization. In high-density, sedentary populations, an individual’s valuation of members of different groups is often guided by generalizations (Tooby et al., 2006), extended to those that share the same ethnic markers (Smaldino, 2019). In short, ethnic groups, and thus intergroup relationships, likely became prevalent during the Pleistocene as sedentism became more prevalent (Sterelny, 2016), perhaps as recently as 130,000 years ago (Singh & Glowacki, 2022). The use of religion as a marker of group identity likely came later, between 50,000 and 100,000 years ago (Sterelny, 2018). However, the timing of the emergence of intergroup and long-distance relationships remains an active area of research.

### How Might the Choice of Cooperative Partners Integrate Information about Location and Group Membership?

A person’s location and group membership are just two of the many criteria that humans weigh when choosing social ties. Individuals often prefer partners with reputations for cooperativeness (Barclay, 2013; Smith & Apicella, 2020), generosity (Smith & Apicella, 2020), and being a “good person” (Pisor & Gurven, 2018). Beyond cooperativeness, individuals often consider whether candidate partners have the means to help (Smith & Apicella, 2020)—e.g., if they hold wealth (Pisor & Gurven, 2018). Additionally, individuals must consider what the market looks like—they must consider what alternative partners are available (Barclay, 2013). Group identity and location are thus just two additional criteria on which individuals may base decisions about social ties (Pisor & Gurven, 2018; Pisor & Surbeck, 2019). Importantly, however, intergroup and long-distance relationships *change the market* by increasing an individual’s number of prospective partners; this may lead to trade-offs in investment in close-distance versus long-distance partners, or in same-group versus intergroup partners—a phenomenon that requires further research (Pisor & Jones, 2021).

## Differentiating Intergroup Relationships and Long-Distance Relationships: a Case Study

Intergroup relationships and long-distance relationships have some nonoverlapping functions and may have emerged at different times in human evolutionary history. However, because intergroup relationships are a primary focus of research on human sociality, long-distance relationships can be easily overlooked, or intergroup relationships and long-distance relationships conflated—as author ACP can attest. While investigating whether opportunities for increased resource access favor intergroup relationships and reduce expression of parochial altruism, ACP came to recognize the importance of distinguishing between intergroup relationships and long-distance relationships.

To illustrate this distinction, we relate ACP’s experience studying the two in rural Bolivia. While collecting survey, economic game, and ethnographic data among three horticultural populations in 2014–2015 (*n* = 200), ACP found substantial variation in how ethnic and religious group boundaries impacted participants’ preferences for forming social ties. Simultaneously, her ethnographic data suggested that long-distance relationships played important roles in the lives and social networks of rural Bolivians. However, ACP found that capturing preferences for long-distance relationships was challenging. A forced-choice task (*n* = 120) deployed with two of the three populations in 2017 underscored the relevance of multiple criteria in partner choice, including indicators of willingness and ability to cooperate, but participants did not express a preference for long-distance partners, likely owing to methodological design.

This case study offers a cautionary tale for scholars studying human sociality in non-WEIRD populations (Henrich et al., 2010): quantitative survey tools that (purportedly) work well in one context may not work in another (Hruschka et al., 2018). The failure of results to generalize across protocols is only apparent when researchers deploy multiple methods to assess the same question.

### Ethnic Groups, Religious Groups, and Twenty-First-Century Rural Bolivia

ACP first decided to study intergroup relationships in Bolivia because of the effects of recent changes in government policy. In the 1980s, 1990s, and early 2000s, Indigenous groups in Bolivia banded together in large-scale movements pushing for Indigenous rights. At long last, in the late 2000s, the Bolivian central government came to recognize the sovereignty of 36 different *pueblos indígenas*.^1^ Despite the rights newly extended to Indigenous people, the state preferentially allocates its limited funds to Indigenous communities that are also *originarios*—living on their traditional lands. Because of this, what was a shared identity of indigeneity in the 1980s–2000s has now splintered as different Indigenous communities, originarios and otherwise, compete with one another for government resources and recognition (Fontana, 2014).

Religious denominations can also delineate group boundaries in rural Bolivia. Evangelical churches of various denominations are expanding their presence in Bolivia (Gill, 1993), as elsewhere in Latin America (Stoll, 1990): as of 2019, approximately 18% of Bolivians identified as “Christian” or “Evangelical” rather than “Catholic” (Pagina Siete, 2019). Rural Bolivians candidly contrast Catholic and Evangelical beliefs, distinguishing what “we” do from what “they” do.

In the midst of these changes to ethnic and religious identities, market participation is on the rise among rural Bolivians. Households increasingly rely more on cash income and less on subsistence production to fulfill their needs (Gurven et al., 2015; Reyes-García et al., 2010). With increased market participation comes increased mobility, contact with middlemen, and exposure to individuals of other pueblos indígenas and individuals who live far away (Pisor & Jones, 2021).

Adopting the assumption that parochial altruism structures intergroup relations, ACP set out to study whether variation in the incentives for intergroup relationships—in Bolivia, between pueblos indígenas or across the Catholic/Evangelical divide—could affect preferences for intergroup relationships. She predicted that individuals with fewer resources might show more interest in building intergroup relationships in order to gain resource access (Pisor & Gurven, 2016, 2018). This prediction was partially supported. However, ACP failed to appreciate the distinction between intergroup relationships and long-distance relationships.

### Collaborating Communities

Three populations of Bolivian horticulturalists—the Mosetén people, the Tsimane’ people, and the Interculturales—are the focus of the present study. The Mosetén and Tsimane’ are pueblos indígenas, recognized by the Bolivian government as originarios because they live on their traditional lands. Mosetén and Tsimane’ have lived in the lowlands for centuries (Godoy, 2015; Huanca, 2008), and the two groups were once a continuous, intermarrying population (Bert et al., 2001; Godoy, 2015; Gurven et al., 2007; Ringhofer, 2010; Sakel, 2011).

Today, however, their lives are quite different. The Mosetén were missionized by Franciscan Catholics during the nineteenth century (Godoy, 2015; Mamani & Huasna Bozo, 2010; Nordenskiöld, 2001), whereas the Tsimane’ were missionized in the twentieth century by evangelical Christians (Huanca, 2008). Efforts by missionaries resulted in access to roads and secondary education for most Mosetén communities by the year 2000 (Pisor & Gurven, 2018); in contrast, only a minority of Tsimane’ communities have access to major roads or secondary schooling (Ringhofer, 2010). Mosetén have more years of education, participate more in the market economy, and have higher mobility than do Tsimane’. Today, whereas Mosetén speak fluent Spanish, the lingua franca of Bolivia, and intermarry extensively with other pueblos indígenas (Pisor & Gurven, 2018), only 14% of Tsimane’ speak fluent Spanish (Pisor & Gurven, 2016) and few have intermarried with other pueblos indígenas. That said, Tsimane’ individuals maintain far-flung connections with other Tsimane’, solidified through visitation, migration, and marriage.

The Interculturales are also a community of Indigenous descent; however, they are not considered originarios and accordingly are not eligible for special government recognition and resources. The word “intercultural” is a designation used by the Bolivian government to recognize communities who are no longer on their traditional lands or who are composed of members of different pueblos indígenas (Albó & Suvelza, 2007). The Intercultural community discussed here is composed primarily of descendants of the Aymara and Quechua pueblos indígenas. These groups were incentivized to move to the area by government relocation programs in the 1950s–1960s and by booms in the logging and quinine industries (Pisor & Gurven, 2016, 2018). Upon arrival, many Interculturales learned horticulture for the first time, sometimes by copying Mosetén. However, they retained many Aymara-influenced institutions, especially with respect to social and political organization. Interculturales are more reliant on the market economy than are Mosetén: they have had reliable access to roads for 25 years longer, and they began to build economic relationships with middlemen much earlier (Pisor & Jones, 2021). On average, Interculturales have as much education as Mosetén, but slightly higher incomes, more market possessions, and higher mobility.

## Measuring Interest in Intergroup Relationships

Consistent with her expectations that parochial altruism structures intergroup relations and that group boundaries would be especially salient in Bolivia, in 2014–2015, ACP and Michael Gurven set out to study the predictors of preferences for intergroup relationships. They used an economic game that they designed—the non-anonymous giving game (NAGG)—to assess how much participants preferred in-group partners at the expense of out-group partners and vice versa (Pisor & Gurven, 2016, 2018; Pisor & Ross, 2022). ACP presented each participant with photos of six strangers^2^: three from the same pueblo indígena or religious affiliation and three from a different one.^3^ She told participants the first name, age, and either the pueblo indígena or religious affiliation of each stranger. ACP then placed three coins (each worth 0.14 USD; total stakes: one-third of a day’s wages) on each photo and three coins in front of the participant. She indicated that the participant could move any coins they wished, or leave the coins as they were, and that any coins left on a photo would be given to the corresponding person in the participant’s name, whereas any coins that the participant left in front of themselves would be theirs to keep. Such games, in which participants are not asked to think about existing real-world relationships or situations, provide more insight into participants’ private preferences—how they would behave if they could. In this case, the game measured preferences for a generous transfer in an experimental context, rather than real-world behavior (Pisor et al., 2020), a point we return to below. For further details on the NAGG, see Pisor and Gurven (2016, 2018).

**Fig. 1:**
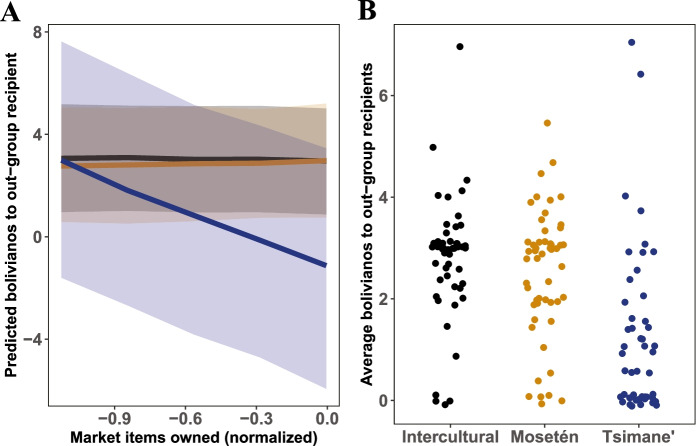
**(A)** Predicted bolivianos (the local currency in Bolivia) given by a participant to an out-group stranger as a function of the total estimated value of market items owned by the participant, normalized relative to other participants in the same population (Pisor & Gurven, 2016, 2018). The shaded areas are the 90% credible intervals. Predictions for Intercultural participants are in black, Mosetén in orange, and Tsimane’ in blue. For details on this model, see ESM §1.3. (**B**) Average bolivianos given by a participant to an out-group stranger (that is, an individual from another pueblo indígena or with a different religious affiliation) in the NAGG (Pisor & Gurven, 2016, 2018). The initial allocation was 3 bolivianos per recipient. Points are jittered

Consistent with their prediction that interest in resource access would increase interest in intergroup relationships, reducing parochial altruism, ACP and Gurven found that Mosetén, Intercultural, and Tsimane’ participants who felt that they were less-well-off than others in their community were more likely to give money to out-group strangers (Pisor & Gurven, 2016). Similarly, Tsimane’ participants who had fewer market possessions than other Tsimane’ participants also gave more to outgroup strangers (Fig. 1a). Though participants gave more to in-group members than to out-group members on average, mean out-group giving was far from zero: 82% of participants gave at least some money to out-group members (Fig. 1b). That said, participants were not uniform in their out-group giving.

First, Tsimane’ preferences looked quite different from Mosetén and Intercultural preferences. Tsimane’ participants were far less likely to give any money to individuals of other pueblos indígenas or religious affiliations (Fig. 1b).^4^ It is possible that Tsimane’ participants valued the money in the game more: Tsimane’ have less wealth on average than Mosetén or Interculturales and see themselves as “have-nots” relative to other pueblos indígenas (Pisor & Gurven, 2018; Pisor & Ross, 2022).

Second, participants used qualities other than a recipient’s group membership in decision-making. After completing the game, participants—especially Mosetén and Intercultural participants—described inferring recipient characteristics from their photos, including their relative need and whether they were a good person, and using those characteristics to make decisions about giving (Pisor & Gurven, 2018; Pisor & Ross, 2022; Pisor et al., 2020).

These differences in generosity toward out-group members are perhaps unsurprising: Tsimane’ have less exposure to members of other pueblos indígenas than do Mosetén or Interculturales. While some of this reduced exposure among Tsimane’ is due to constrained mobility (given the few passable roads and the expense of gasoline for river travel) and limited access to education (there are few fluent Spanish speakers), some is also due to active avoidance of out-group members. At the time of European contact, Tsimane’ were well-known among other Indigenous groups in the region as salt traders (Godoy, 2015; Nordenskiöld, 2001). However, their interactions with highland Bolivians (*collas*) and non-Indigenous lowland Bolivians (*cambas*) have been marked by misunderstandings, marginalization, and discrimination; Tsimane’ have retreated from contact with the Spanish and *cambas* when these groups took advantage of them (Godoy, 2015; Huanca, 2008; Ringhofer, 2010).

## Conflating Two Questions

Although market participation is increasing for all three populations, none are interacting with other pueblos indígenas for the first time. As mentioned above, Tsimane’ were renowned salt traders at the beginning of the twentieth century: salt in the Amazon is heavily concentrated in certain areas (Reeve, 1993), creating a demand for long-distance trade. Mosetén have long traded with lowland groups for tools, medicine, and plants (Lathrap, 1973; Ringhofer, 2010) and, centuries ago, traded with the Inka for metal goods (Godoy, 2015). Before Columbus, Quechua and Aymara, whose descendants are residents of the Intercultural community, had trade networks spanning the Andean ecozones, ensuring access to foods from different regions (Klein, 2011). Importantly, members of these networks were often members of the same ethnic group.

The common characteristic of these connections is not that they span group boundaries, but that they span distance. ACP had assumed that intergroup relationships were consonant with distance. However, it is long-distance relationships, not intergroup relationships, that are especially likely to help individuals diversify their resource access across geographic space—different groups may live close together, for example, as Interculturales and Mosetén do. ACP had not even *thought* to investigate whether individuals with less resource access would be more interested in long-distance relationships. Once she realized her oversight, she returned to the Mosetén and Intercultural communities in 2017 to revisit how individuals were using social connections to access nonlocal resources—this time using methods that she hoped would distinguish between preferences for intergroup relationships and long-distance relationships.

## The Reality of Long-Distance Relationships

Though long-distance relationships can function both to buffer local shortfalls and to provide access to resources not locally available (as discussed above), ethnographic and survey evidence suggest that for Interculturales and Mosetén, they are more important for accessing unavailable resources than buffering shortfalls. In 2014, much of lowland Bolivia was hit with severe flooding. Landslides destroyed roads serving Mosetén and Intercultural communities and cut power and cell service for more than a month. Because floods are one of the most common natural disasters in Bolivia (World Bank, 2020), both Mosetén and Interculturales have cultural practices for managing the risk of resource shortfalls due to flooding—for example, both raise pigs and chickens that can be slaughtered during hard times, and Interculturales have a system of loaning rice. In 2014, families ate their pigs, chickens, and rice. Once these fallback foods were depleted, Mosetén marched down their destroyed road to demand support from the municipal and federal governments. Both the Intercultural and Mosetén communities eventually used motorized canoes to ferry emergency supplies—mostly food and medicine—from the local town. When waters receded, the municipal government sent equipment and laborers to repair the roads. Mosetén and Intercultural families who could not absorb the cost of their destroyed crops sought loans from local banks.

**Fig. 2:**
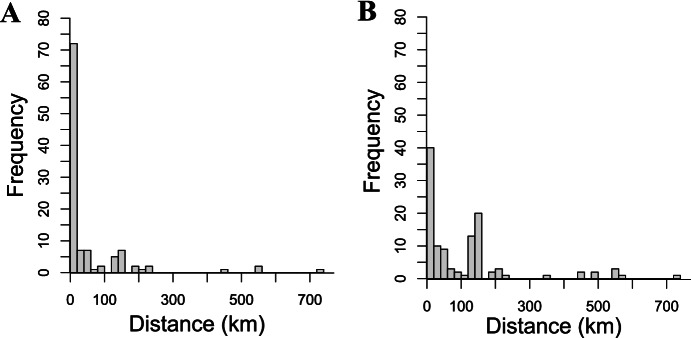
Each participant was asked to name someone who could help them (**A**) with a loan of 500 bolivianos ($70, or 8 days’ wages) if a flood destroyed their crops, and (**B**) find “a good job that pays well.” Most participants named individuals, but some named government entities, organizations, or private companies. Counts reflect the distance between the participant and where these named individuals or entities were located

Long-distance relationships were not central to household responses to the 2014 flood. Because landslides had severed roads and communications, individuals could not contact their long-distance connections. Instead, resources that could buffer the local shortfall came from the government and from banks. This is not an unusual pattern: in many rural contexts, government entities, international organizations, and banks can take the place of social connections for managing risk (Pan, 2009). Given the limited role of long-distance relationships in 2014, it is perhaps unsurprising that three years later, when asked who would help them with a loan during a hypothetical flood, Mosetén and Intercultural participants were more likely to name same-community individuals or government entities than connections living at a distance (Fig. 2a) (Pisor & Jones, 2021).

Though they may not be an important source of risk buffering for Mosetén and Interculturales, long-distance relationships are crucial for access to resources not locally available (Pisor & Jones, 2021). First, both communities rely on middlemen—often based in the capital city of La Paz (seven hours away by car)—to purchase their crops. Second, both increasingly engage in migrant labor to supplement their incomes. Long-distance relationships are key to finding work as a migrant laborer. For example, when asked who they would contact for a “good job that pays well,” 65% of participants named an individual or entity outside of their community (Fig. 2b). Third, market participation provides cash income, which translates into increased mobility. Mosetén and Interculturales increasingly have business in La Paz and send their children to university or job training programs there. Individuals report that La Paz residents help them navigate local bureaucracy, from completing government paperwork to enrolling in universities. Further, given the high cost of lodging in La Paz, city residents can provide low-cost shelter. Fourth, long-distance relationships provide access to goods only available in the city. For example, La Paz residents frequently send parcels, or *encomiendas*—which can contain anything from bread to cell phones or televisions—by bus to rural residents.

The Mosetén and Interculturales have various means for maintaining their long-distance relationships (Pisor & Jones, 2021). Reciprocal exchanges of encomiendas are common: residents of La Paz often request fresh produce such as plantains and mandarin oranges, which are expensive in the city. Community members also send and receive money transfers, or *giros*; giros may be used to reimburse someone for an expensive encomienda or to loan money. Semi-reliable cell phone service has been available to the Mosetén and Interculturales since 2010. Phone calls and, increasingly, WhatsApp or Facebook Messenger are used to maintain contact with long-distance relationships. Visitation remains important to relationship maintenance as well: depending on an individual’s means, they may take buses, shared taxis, or their own vehicles to visit long-distance relationships, often for several days.

### Who Are These Long-Distance Connections?

Intercultural and Mosetén community members maintain long-distance relationships with both relatives and nonrelatives. Unsurprisingly, many are consanguineal or affinal kin (Pisor & Jones, 2021). However, Mosetén and Intercultural Catholics also strategically use fictive kinship—namely, godparent relationships (*compadrazgo*)—to solidify long-distance relationships with individuals they believe are wealthy or influential enough to help them or their children (Hubbard & Pisor, 2022; Mintz & Wolf, 1950). Individuals who are from La Paz and spend time both in the community and in the city are favorite choices, especially teachers, doctors, and middlemen. Long-distance relationships are also forged during periods of temporary migration: for example, during stints of migrant labor, while studying at university or in career programs, or, for men, while completing military service.

When individuals have long-distance relationships with non-kin, however, they often do not know the pueblo indígena of their social partner. In 2017, ACP asked participants to identify the pueblo indígena of their long-distance relationships. Participants had a difficult time understanding the question; ACP often cycled through several phrases—pueblo indígena, *descendencia* (descent), *parentezco* (kinship)—or gave examples before a participant was able to answer. Once they understood the question, participants often guessed when responding. Some identified all friends from the lowlands as *cambas*, even though Indigenous peoples are also from the region; others reasoned that if someone lives in La Paz, they must be Aymara, the dominant pueblo indígena in the city.

In short, the pueblo indígena of a long-distance relationship was far less salient to participants than might be expected given the political landscape in Bolivia. It was also far less salient than we might predict given how much sway ethnicity is given in the evolutionary anthropology and evolutionary psychology literatures. See Moya and Boyd (2015) for a similar example from Perú.

## Focusing on Intergroup Relationships Can Mask the Importance of Long-Distance Relationships

Given the wealth of ethnographic and self-report evidence that long-distance relationships are important to Mosetén and Interculturales, ACP set out to design a task that could potentially distinguish participants’ preferences for long-distance relationships from their preferences for intergroup relationships. Though individual-level differences in resource access may predict differential investment in long-distance relationships, ACP was also aware that participants with less wealth may have had more incentive to keep money for themselves in the 2014–2015 economic game. She reasoned that a task not involving money might reveal preferences for forming new social relationships independently of preferences for giving or keeping money. Drawing on marketing research, she chose a paired-comparison forced-choice task (see Rao et al., 2014) to assess which traits participants preferred in candidate friends.

ACP presented participants with pairs of cards representing hypothetical individuals, each described by six categories of characteristics (ESM §1, Fig. S1), and asked which of the two cards they would prefer as a new same-sex friend. In addition to the location of the candidate friend, their pueblo indígena, and their religious affiliation, three other categories of characteristics were included—trustworthiness, being a “good person” (*buena gente*), and wealth—since these characteristics were some of the most frequently mentioned during the NAGG game played in 2014–1015 (Pisor & Gurven, 2018).

**Fig. 3:**
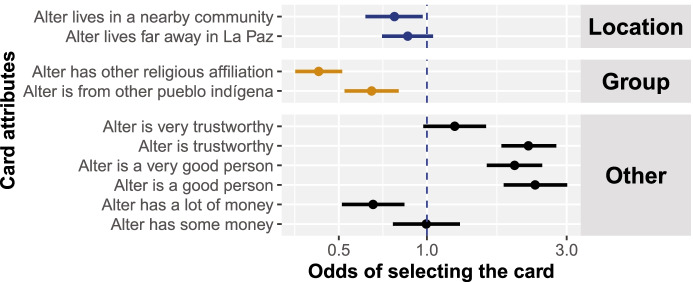
Each participant (*n* = 125) was presented with a pair of hypothetical individuals and was asked which they preferred as a new friend. Each evaluated 18 pairs of individuals. Here, we present nonstandardized estimates (means and 90% credible regions) from a logistic regression; these estimates reflect the odds of selecting one individual if it were to differ by only a single attribute from the other individual. The base case is a candidate friend who lives in the same community, is from the same pueblo indígena and has the same religious affiliation, and who is not good, not trustworthy, and has no money. The estimates indicate that participants preferred a same-community friend over more distant friends, same-ethnic- and same-religious-group friends over those from other groups, and friends that are “good people,” trustworthy, and—interestingly—not excessively wealthy

Participant preferences in the choice task, however, did not reflect the prevalence of long-distance relationships in these communities discussed in the previous section. Participants preferred candidate friends of their own pueblo indígena and religious affiliation (Fig. 3). They also had a slight, though inconsistent, preference for friends from their same community over friends from other places.

Why the discrepancy between the behavioral data reported above and the preferences elicited by the choice task? Some participants reasoned aloud during the decision-making process, providing insight (Bernard, 2017). These participants would often identify one characteristic of the six that stood out to them (“this one is from my church, so I pick him”) and continue to make decisions based on that criterion across all pairs of cards. In other words, even though characteristics of candidate friends varied across cards, participants stopped attending to characteristics other than the one they initially selected.

Not only did participants’ preferences in the choice task not reflect the real-world importance of long-distance relationships, they did not reflect the preferences elicited by the 2014–2015 NAGG game either. Recall that in the game, despite some preference for in-group members, there was substantial out-group giving. In total, 63 participants completed both the 2017 choice task and the 2014–2015 game. There was no relationship between participants’ preferences in the 2014–2015 game and their preferences in the 2017 choice task (see ESM, Fig. S3). Perhaps this reflects real changes in preferences over time—possibly related to changes in political climate or in material wealth. However, it is likely that differences in methodological design also play a role.

### Methodological Design: a Cautionary Tale

There are several reasons why participants may have used pueblo indígena membership as a rule of thumb for choosing social partners in the choice task. First, rules of thumb can ease the cognitive burden of repeated decision-making. If pueblo indígena membership was made salient by the design of the task, participants may have first looked for differences in membership between the two candidate friends and, if a difference was present, used it for decision-making (i.e., a “take-the-best” heuristic; Gigerenzer & Goldstein, 1996). Participants may especially lean on heuristics when they find tasks tedious (e.g., Tucker, 2017), as they did in this study.

Second, with so few categories of friend qualities (here, six total), demand characteristics or experimenter effects are a concern. Perhaps participants intuited that ACP was interested in group membership, especially since that was how she framed her research in 2014–2015 when talking to community members (Nichols & Maner, 2008). In other words, if researchers are strongly focused on group membership, they can inadvertently increase the salience of group boundaries—even for groups that are not particularly salient locally—and thus alter their research findings.

Third, when features of the real world are removed and partner choice is based only on a list of characteristics, group boundaries may be more salient than they are in real-world partner choice. Cueing identity can shift elicited preferences (Benjamin et al., 2010).

Fourth, there is not a one-to-one mapping between preferences and behavior (Pisor & Ross, 2022; Pisor et al., 2020). Even if this task were measuring what it was designed to measure—and the foregoing gives us reason to doubt this—participant preferences can be eclipsed by real-world constraints and incentives, leading to a disconnect between “cheaply elicited” preferences and behavior in the real world.

For similar discussions of how preferences elicited by experimental tasks can differ from the real world, and why field researchers should use multiple methods to triangulate the reality of preferences and behavior, see Tucker (2017), Hruschka et al. (2018), and Pisor et al. (2020).

## Discussion

Intergroup relationships and long-distance relationships are two different features of human sociality with partially overlapping functions. Given the high costs of intergroup conflict and its sequelae, researchers often focus their attention on how intergroup relationships can defuse conflict—usually between ethnic groups. However, intergroup relationships and long-distance relationships may have emerged at different points in human evolutionary history and may be products of different selection pressures. Long-distance relationships can span distance without crossing group boundaries, providing access to resources that are not available to an individual’s local social partners or that are less correlated with locally available resources. While intergroup relationships can also offer access to resources not locally available, especially when different groups specialize in producing different resources, intergroup relationships are distinct in that they can also be strategically used to reduce intergroup conflict. In short, researchers can ask separate questions about intergroup relationships and long-distance relationships, including why one may be common in a given context while the other is not.

Drawing on a case study from Bolivia, we illustrated the importance of distinguishing intergroup relationships from long-distance relationships. Since ethnic groups, or pueblos indígenas, are central to contemporary Bolivian discourse and compete with one another for government resources, ACP expected that in rural Bolivia, ethnic group boundaries would be salient, parochial altruism common, and any intergroup relationships noteworthy. However, in three populations located within 50 miles of each other, intergroup relationships varied in both their prevalence and their salience.

For Tsimane’, long-distance relationships are more common than intergroup relationships. The area most Tsimane’ inhabit is large (the land to which they hold title alone measures 4,013,228 square kilometers; Ringhofer, 2010), population density is low, and few non-Tsimane’ live in Tsimane’ territory. Accordingly, strangers, whether in-group or out-group, live at a distance and long-distance relationships with other Tsimane’ are common. However, the distance between Tsimane’ and other ethnic groups is not an exogenous variable: due to a long history of inequality and discrimination, Tsimane’ have retreated from contact with other groups (Godoy, 2015; Huanca, 2008; Ringhofer, 2010). Perhaps unsurprisingly, in an economic game designed to gauge interest in forming new relationships, Tsimane’ participants preferentially invested in Tsimane’ strangers—by definition, individuals living at a distance—rather than strangers from another ethnic group (Fig. 1; Pisor & Gurven, 2016, 2018).

In contrast, in a Mosetén community and an Intercultural community, intergroup relationships are ubiquitous and long-distance relationships very common. Both communities are also rural, so strangers live at a distance; however, mobility and intermarriage between ethnic groups are much higher than among Tsimane’. Accordingly, for Mosetén and Interculturales, the discrepancy between in-group and out-group giving in the non-anonymous giving game was less than was observed among Tsimane’, consistent with the pervasiveness of intergroup relationships (Fig. 1; Pisor & Gurven, 2016, 2018). Long-distance relationships are ubiquitous too—important for selling crops, finding jobs, and navigating the capital city, from getting lodging to managing bureaucracy. Asked to name who they would contact for a “good job that pays well,” 65% of participants named an individual or entity outside their community (Fig. 2b).

As we have highlighted here, researchers should not be surprised that preferences for intergroup relationships vary across participants and communities. First, despite focus on ethnic groups in evolutionary approaches to human behavior, not all groups are ethnic groups (Pisor & Ross, 2022); if ethnic group boundaries do not structure cooperation—as is largely the case for Mosetén and Interculturales, but notably *not* for Tsimane’—they will be less salient to individuals (Moya & Boyd, 2015). Second, valence and valuation matter; for Tsimane’ participants, intergroup interactions are often characterized by a negative valence—given past experiences of misunderstandings, marginalization, and discrimination—and the expected value of intergroup ties is likewise low. In general, we should expect an individual’s interest in intergroup relationships to track the expected net benefits of such relationships (Pisor & Gurven, 2018). These net benefits will be impacted by the relevance of group boundaries for cooperation and by past experiences with out-group members, among other criteria. Likewise, the expected net benefits of long-distance relationships should influence partner choice; we expand on this below.

### Long-Distance Relationships: Improving Methodological Design

Though ACP designed a task to tease apart partner preferences based on group membership and location, she found that ethnographic data on long-distance relationships were surprisingly hard to corroborate through simple experimental tasks. The forced-choice task did underscore that partner choice is often based on characteristics pertinent to cooperation, such as willingness or ability to help (Barclay, 2013; Pisor & Gurven, 2018; Smith & Apicella, 2020)—location is just one of many partner-choice criteria. It also underscored that the salience of group membership can be primed by empirical methods (e.g., Pisor et al., 2020), as suggested by the lack of correspondence between participants’ allocations in the non-anonymous giving game and their decisions in the forced-choice task.

Our case study serves as a cautionary tale, with lessons for how researchers can better measure long-distance relationships going forward. Given that long-distance ties are often not as salient as local ties (Wobst, 1978), asking about them directly is more fruitful than hoping they will turn up in experimental tasks with little demonstrated external validity. ACP, her collaborators Kristopher Smith and Monique Borgerhoff Mulder, and their team ask about long-distance friendships directly in their ongoing research in Tanzania; thus far, they find this approach appears to have good face validity—that is, it appears to actually measure what it is supposed to measure (Smith et al., 2022).

### Long-Distance Relationships: Future Directions

As we demonstrated here, although intergroup relationships and long-distance relationships have overlapping functions, they are distinct types of relationship and vary in their prevalence, even between nearby communities. The focus on intergroup competition in the social sciences tends to make intergroup relationships noteworthy and to mask other features of human sociality, such as the importance of long-distance relationships in human social life over the past 300,000 to 1 million years (Foley & Gamble, 2009; Layton et al., 2012).

To better characterize the structure and importance of long-distance relationships, we recommend the following avenues for further research:

- *Where and when will long-distance relationships be more common?* As outlined here, we should expect long-distance relationships to be more common where and when (a) resource shortfalls can strike entire communities and (b) key resources cannot be obtained locally. Long-distance relationships can be especially important when shortfalls regularly strike entire communities—when shortfalls recur (Pisor & Jones, 2021)—and when government support is absent or unreliable (Pan, 2009). Climate variability can increase the frequency and duration of shortfalls, so in the absence of sufficient government support, we can expect long-distance relationships to become even *more* important given contemporary anthropogenic climate change (Jones et al., 2021; Pisor & Jones, 2021). With respect to nonlocal resource access, archaeological data offer a classic example of long-distance relationships: artifacts provide evidence of how nonlocal resources were obtained through long-distance exchange networks (Braun & Plog, 1982; Irwin et al., 2019; Lathrap, 1973; Spielmann, 1986). Even in locations where online platforms offer fast access to nonlocal resources, from job postings to at-home delivery, long-distance relationships continue to be a crucial source of fiscal resources (e.g., remittances or loans) and word-of-mouth information (Pisor & Surbeck, 2019; Smith et al., 2022).
- *How are long-distance relationships maintained? Do they have the same characteristics as short-distance relationships?* Research on long-distance relationships has traditionally focused on relationships formed when two individuals live in the same place, and then one moves away (Policarpo, 2016). Long-distance relationships are often characterized by commitment, mutual trust, and seeking the advice of one another; however, compared with short-distance relationships, commitment to long-distance relationships varies more across time (Johnson et al., 2009). But what about long-distance relationships forged between two individuals who have *never* lived in the same place—do individuals pick long-distance friends using the same criteria they use to choose short-distance friends? Further, do the strategies individuals use to maintain long-distance relationships forged from a distance look like those used to maintain relationships that start local and become long-distance relationships? We are tackling these questions in ongoing research.
- *What are the consequences of long-distance relationships for cooperation?* Existing theoretical work suggests that individuals may penalize in-group members for forging intergroup relationships if these endanger the effectiveness of within-group cooperation (Bowles, 2008). However, when an individual forges a relationship that spans group boundaries (Fearon & Laitin, 1996) or distance (Fitzhugh et al., 2011), this can also generate benefits for in-group members or neighbors. Consider, for example, the cooperation required to manage extensive common-pool resources such as fisheries, watersheds, grasslands, and forests, accessible by multiple communities. To avoid depleting these resources, members of the communities must not only avoid overharvesting, they must also coordinate in setting and enforcing management rules (Varughese & Ostrom, 2001). Are individuals with long-distance relationships that span the boundaries of these communities less likely to overharvest and more likely to actively contribute to resource management? ACP, Smith, Borgerhoff Mulder, and colleagues have research underway designed to answer this question.

## Conclusion

Intergroup relationships are a central focus of social science research, and rightly so: intergroup relationships offer a solution to the high costs of intergroup conflict (Brewer, 2010; Dovidio et al., 2003; Pettigrew & Tropp, 2006; Riek et al., 2006). However, studying intergroup relationships to the exclusion of long-distance relationships, or even conflating the two, means that our research questions and data analysis may be imprecise, and our characterization of human sociality may thus be incomplete. We have presented a case study from rural Bolivia that emphasizes this point: after initially conflating intergroup relationships and long-distance relationships, ACP realized that long-distance relationships were more crucial to securing nonlocal resource access than were intergroup relationships. Long-distance relationships were also more difficult to study using standard quantitative methods; the mixed-methods findings we presented here underscore the importance of field-validating the methods researchers use to study human sociality.

The importance of studying long-distance relationships is not limited to the theoretical. If archaeological, ethnographic, and historical data are any indication, in the absence of sufficient government support, long-distance relationships may become an increasingly central part of individuals’ responses to climate change (Jones et al., 2021; Pisor & Jones, 2021). Additionally, long-distance relationships may improve the effectiveness of management for large natural resource areas accessible by multiple communities. In sum, improving our understanding of when and how contemporary peoples use long-distance relationships has broad relevance.

## Supplementary Information

Below is the link to the electronic supplementary material.

Supplementary file1 (PDF 133 KB)

## Footnotes

1 *Pueblos indígenas* are specifically defined as Indigenous groups whose members are *originarios*—living on their traditional lands—and whose members share cultural institutions—explicitly recognized by the government as their *usos y costumbres*. This definition makes “pueblo indígena” akin to the definition of “ethnicity” used in the evolutionary anthropology literature (e.g., Bunce & McElreath, 2017; McElreath et al., 2003; Moya & Boyd, 2015).

2 Research assistant Bernabe Nate Añes was integral to data collection among the Tsimane’.

3 In this rural context, strangers by definition live at a distance; because communities rarely exceed 1,000 people, local individuals are known.

4 Note that at the time of data collection, most Tsimane’ were affiliated with the same evangelical church, such that recipients of the same religious affiliation were frequently also Tsimane’—and Tsimane’ participants could easily tell who was Tsimane’ from looking at the photos (Pisor & Gurven, 2018).
