# Supplementary material for: Distinguishing Intergroup and Long-Distance Relationships
Source: Hum Nat. 2022 Oct 1;33(3):280–303. doi: 10.1007/s12110-022-09431-1 (PMC9741575; doi:10.1007/s12110-022-09431-1)
Supplement: Supplementary file 1 — Supplementary file1 (PDF 133 KB) [file 12110_2022_9431_MOESM1_ESM.pdf]

Electronic Supplementary Material (ESM) for: Distinguishing intergroup and long-distance relationships

Anne C. Pisor[1,2] and Cody T. Ross[2]
[1] Department of Anthropology, Washington State University.
[2] Max Planck Institute for Evolutionary Anthropology. Dept. of Human Behavior, Ecology and Culture. Germany.
Human Nature 33(2), 2022, https://doi.org/10.1007/s12110-022- 09431-1

Contents

1 The choice task **1**
1.1 Data collection 1
1.2 Data preparation 1
1.3 Statistical modeling 2
1.4 Game play and the choice task 3

2 Results **4**
2.1 The effects of card characteristics on card choice 4
2.2 The effects of economic game play on card choice 4

Data, metadata, and code for this study are available at `www.github.com/annethro/parochialism`

1. The choice task

1.1. Data collection

Participants (n=120; 40% female) were interviewed by ACP and two research assistants in April–June 2017. Participants received monetary compensation for their time. Study protocol were approved by the communities and the Mosetén tribal organization. Given mixed literacy but familiarity with signing forms, participants were read a consent form and provided their written consent via signature. All field protocols were approved by the Max Planck Institute for Evolutionary Anthropology Department of Human Behavior, Ecology, and Culture, and declared exempt from additional IRB oversight.

ACP presented participants with pairs of cards representing hypothetical individuals—each described by six categories of characteristics (Figure S1). The levels for these six categories were as follows (translated from Spanish):

- **Location:** this community, the other side of the river valley, La Paz
- ***Pueblo indígena***: Mosetén, Tsimane', Aymara, Quechua, Trinitario
- **Religious affiliation**: Catholic, Evangelical
- **Trustworthiness**: not trustworthy, trustworthy, very trustworthy
- **Good person**: not a good person, good person, very good person
- **Wealth**: does not have money, has money, has lots of money

ACP generated the complete orthogonal array of cards for these six categories and their levels using a Latin square design such that each card in the array differed by at least one level on one of the six categories. ACP randomly sampled 180 cards from this array and randomly sorted them into 90 pairs (without replacement; cf. Rao et al. (2014)) using the mix-and-match method in the R package `support.CEs` (version 0.4.1; Aizaki (2012)). These pairs were then randomly assigned to five blocks; within each block, the pair was randomly assigned an order (1-18) and one card was randomly assigned to appear on the left. To control for any effects of the order of presentation of the six categories, the order of the six categories was randomized into two versions of each block, creating 10 total versions of the task (e.g., Version 1, from the top of the card to the bottom: religious affiliation, trustworthiness, pueblo indígena, location, good person, and wealth (Figure S1); Version 2: trustworthiness, wealth, religious affiliation, good person, location, pueblo indígena). Each participant was presented with one of 10 versions of the choice task and made 18 sequential decisions between pairs of cards.

1.2. Data preparation

Choice task design, data preparation, and analyses were conducted using R version 4.0.2 (R Core Team 2016).

To reduce participant identifiability, we binned participant ages into 5-year bins (e.g., an age of 36 was

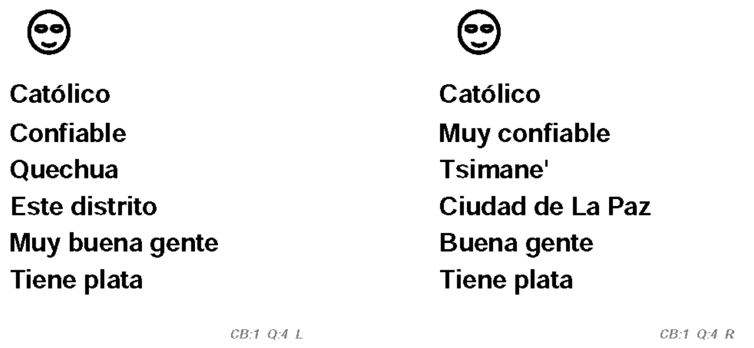

Fig. S1: An example of one of the 18 sequential decisions made by each participant in the paired comparison choice task. The codes at the bottom right indicate that this pair was the fourth presented to participants who completed the first version of the task; the card marked “L” always appeared on the left and “R” always on the right.

rounded to 40; an age of 21 was rounded to 20). To aid in model estimation, we normalized net household income—that is, the participant’s household’s estimated income over the last month, minus their estimated expenditures; years of schooling; and age such that a value of 0 represents the sample mean and a value of 1 represents one standard deviation. We also normalized the average amount given to out-group candidate recipients in the 2014-15 economic game for the models described in section 1.4.

Of the 120 total participants, 13 reported having no religious affiliation and one was not asked about their years of schooling. Given our interest in whether participants are choosing candidate friends based on religious affiliation and our inclusion of years of schooling as a control, we imputed these data using predictive mean matching, implemented with the `mice` package (version 3.10.0; Van Buuren and Groothuis-Oudshoorn (2011)).

1.3. Statistical modeling

We used a Bernoulli mixed-effect model with logit link to investigate which card characteristics predicted a participant’s choice of the right-hand card. Since the choice between two cards in a pair was binary, our decision to model participants’ decisions using the right-hand card was arbitrary; we could just as easily have picked the left-hand card. We parameterized models such that estimates reflect contrasts to lower levels—that is, “in-groups” (i.e., same community, same pueblo indígena, same religious affiliation) and the absence of a quality (i.e., a bad person, someone who is not wealthy, someone who is not trustworthy)—and can be compared to no difference between cards. For example, consider the combinations for Location: Same Valley and Location: City in Table S1.

In Row A, the indices indicate that the candidate friend on the left-hand card was from the same river valley, as there is a -1 (meaning left) in Loc.SameValley_Diff column, not a (right); the candidate friend on the right-hand card was from the city, as indicated by the 1 in the Loc.City_Diff column. By extrapolation, then, neither candidate friend was from the same community. In Row B, the candidate friend on the right-hand side was from the same valley. The value for Loc.City_Diff is 0—indicating that either both candidate friends are from the city, or neither is—but since we know that the right-hand candidate friend is from the same valley (given Loc.SameValley_Diff is 1), that means that neither is from the city and therefore the left-hand candidate friend is from the same community. In Row C, the coding scheme does not reveal where the candidate friends are from, but rather that there is no difference between the left-hand card and the right-hand card: both candidate friends are in the same location, regardless of which location that is. This coding scheme means that parameter estimates for Location tell us whether (i) a participant is more likely to pick the right-hand candidate if they live further away than the candidate on the left-hand side, and (ii) whether there’s something specific about living in the same valley or living in the same city that participants use to make their decision. An alternative coding scheme that tells us just (i), not (ii), is described and implemented in our code for comparison (available at `www.github.com/annethro/parochialism`).

Data are coded such that pueblo indígena and religious affiliation are specific to the participant, based on how they self-identified in census data. For example, imagine a participant who self-identified as Evangelical and Quechua. If she is shown the two cards in Figure S1, this is coded as 1 for pueblo indígena—her own pueblo indígena appears on the left, whereas a different pueblo indígena appears on the right—and coded as 0 for religious affiliation—the same religious affiliation appears on both cards.

Models were implemented with the `brms` package (Bürkner 2017), which passes Bayesian models to Stan (Stan Development Team 2019). We used weakly informative priors. For fixed effects, given parameters in logistic models are on a scale of 0-1 before they pass through the link function, we used a normal prior with a mean of

Table S1: The potential combinations of values for Location: Same Valley and Location:City on two cards.

Row	Loc.SameValley_Diff	Loc.City_Diff
A	-1	1
B	1	0
C	0	0

0 and standard deviation of 1. For random effects, given the traceplots of initial model runs, we used an exponential distribution with a rate parameter of 1 to make exploration of the parameter space more efficient. For a discussion of the choice of priors, see (McElreath 2016).

We fit models both with controls and without. We anticipated that participant age, sex, years of schooling, and household net income over the previous month might affect preferences for a candidate friend who lived at a distance, was part of a different pueblo indígena, or was of a different religious affiliation. For example, because mobility is higher among males than females and, thanks to access to roads, has been higher for a larger proportion of younger individuals' adult lives, sex and age affect exposure to individuals at a distance or from other pueblo indígenas and could impact preferences accordingly (Pisor and Jones 2020). Because the outcome of our models was choosing the right-hand card, treating controls as additive did not make sense: this would control for preferences for choosing right, not preferences for choosing someone who lived at a distance, for example. Accordingly, we fit a separate model for each control, in which we interacted the control with the three predictors of interest: candidate friend location, pueblo indígena, and religious affiliation. As the inclusion of each control did not yield results that were qualitatively different from the model with no controls, we report the model without controls in the main text.

1.4. Game play and the choice task

We analyzed whether play in the Non-Anonymous Giving Game (Pisor and Gurven 2016; **?**)—that is, average amount given to recipients from a different pueblo indígena—in 2014–15 was associated with preferring candidate friends from a different pueblo indígena in 2017; we did the same for average amount given to recipients from a different religious affiliation and preferring candidate friends with a different religious affiliation. We also explored whether out-group giving—regardless of whether the out-group was religious or a pueblo indígena—predicted preferring candidate friends living at a distance.

A subset of 80 participants (50% female) completed both the choice task and the 2014–15 economic game. In 2014–15, before playing the game, participants completed a sorting task where they identified which pueblos indígenas and religious affiliations they identified with the most (position one) and the least (position five). From this, we classified a participant's in-groups as those in the first or second positions, out-groups as those in the fourth and fifth positions, and "intermediate" groups as those in the third position. Of the 80 participants who completed both the choice task and the game, 63 placed groups in the fourth and fifth positions and were thus able to play the economic game with either a religious or pueblo indígena out-group. Because the other 17 did not place groups in the fourth or fifth positions, they played the game with either one in-group and one intermediate group or two in-groups; given our interest in out-group giving, we excluded them from analysis accordingly.

Note that in 2017, we did not complete the sorting task with participants before conducting the choice task; we coded the in-group/out-group status for candidate friends based on whether the card had the participant's pueblo indígena on it (pueblo indígena in-group) or not (pueblo indígena out-group), and whether it had the participant's religious affiliation on it (religious in-group) or not (religious out-group).

To check for a relationship between how a participant played in the economic game and their choices in the choice task, we ran two models, interacting:

1. (i) pueblo indígena in-group/out-group status for candidate friends from the choice task with (ii) participant presented with a pueblo indígena out-group in the game and (ii) average amount the participant gave to each out-group member in the game—a three-way interaction. We did the same for religious affiliation. We report the results from this model in Figure S3, if it helps the reader visualize the interactions.
2. (i) location of candidate friends from the choice task with the amount the participant gave to each out-group member on average—a two-way interaction.

Models 1 and 2 were fit both with and without controls. First, as described in 1.3, we fit one model for each control—age, sex, years of schooling, and household net income over the previous month—in which we interacted the control with the predictors of interest, described in bullet points 1 and 2 immediately above, creating four-way and three-way interactions.

Second, we ran three additional models, each with one of three additional controls. The first was planned: whether the participant chose to give money anonymously in the 2014–15 economic games. (Some participants strongly wished to do so, so ACP allowed them this option; see Pisor and Gurven (2016, 2018) for details.) The second and third were included as exploratory analyses. For backstory, household net income over the previous month was entirely uncorrelated (r = -0.01) between 2014–15 and 2017. This may be because ACP conducted the interviews in spring and summer in 2014–15 vs in

fall in 2017, and cash crop incomes vary substantially by season; however, it may also reflect changes in household circumstances in the intervening two years that could affect preferences. Accordingly, in one model we explored the effects of controlling for household net income in 2014–15, and in the other, controlling for the difference in household income between 2017 and 2014–15.

Model specification was otherwise identical to that described in Section 1.3—including predictive mean matching, with which we imputed data for three people without a religious affiliation and one person missing data on their schooling.

2. Results

Models with and without controls returned qualitatively similar results; we thus report models without controls here and in the main text for simplicity. Again, models estimate the probability of picking the *right-hand* card based on the qualities that appear on the left or on the right, per the coding scheme described in 1.3, and we focus on right-hand estimates accordingly in the main text. For completeness, however, we plot both the probability of picking left and the probability of picking right here in the supplement. All estimates are non-standardized means and 90% credible intervals.

2.1. The effects of card characteristics on card choice

Participants preferred candidate friends who were not from La Paz, the capital city of Bolivia; we can infer this because when the candidate friend on the right-hand side was from La Paz but the candidate friend on the left-hand side was not, participants were more likely to pick the left-hand card (see top box in Figure S2). They also preferred candidate friends who were not from a nearby community—that is, not from the same river valley; they were less likely to pick the right-hand card when it said the candidate friend lived nearby. Taken together, we can infer that participants preferred same-community candidate friends over friends from elsewhere.

Likewise, participants preferred candidate friends from their own pueblo indígena and their own religious affiliation over those from other pueblos indígenas and other religious affiliations (Figure S2). Note that these latter effects are more pronounced than the effect of location—that is, the odds ratios for picking right are lower and for picking left are higher.

Consistent with preferences elicited in the 2014–15 economic game, participants strongly preferred candidate friends who were “good people” or “very good people”—this was the largest effect observed (Pisor and Gurven 2018). Participants also preferred participants who were “trustworthy” or “very trustworthy." Participants avoided picking candidate friends described as having “a lot of money.”

In models including one of each of the controls, older participants were more likely to prefer a candidate friend of the same religious affiliation and participants with higher net incomes were more likely to prefer someone not from the same river valley. Other controls did not predict choice of candidate friend.

2.2. The effects of economic game play on card choice

We next examine whether game play in the 2014–15 Non-Anonymous Giving Game predicts card choice in the 2017 choice task. The posterior means and 90% credible intervals for the predictors of interest are reported in Figure S3. The average amount a participant gave to out-group recipients in the 2014–15 economic game was not related to whether participants chose cards based on out-group membership in 2017—regardless of whether out-group members were from a different pueblo indígena or from a different religious affiliation. Likewise, the average amount a participant gave to out-group recipients in 2014–15 was not related to whether they chose cards based on the candidate friend’s location in 2017.

The 90% credible interval included an odds ratio of 1, suggesting no effect, for a candidate friend living in La Paz, from a different pueblo indígena, and having a lot of money (Figure S3). Note that the difference in the magnitude of these estimates vs the model including only card characteristics (Section 2.1; Figure S2) is unsurprising given the sample size also differs (n=120 in Section 2.1, n=63 in this section).

Keeping in mind this difference in sample size, the inclusion of controls reduced the magnitude of the following effects of card characteristics:

- **Age.** The older the participant, the more they preferred candidate friends not from the same valley or from La Paz, and preferred candidate friends who were very trustworthy and not “very wealthy"
- **Sex**. Male participants preferred candidate friends not from the same valley or fron La Paz if on the left, and not “very wealthy”
- **Years of schooling**. Participants with more years of schooling preferred candidate friends not from the same valley and not from La Paz, but from the same pueblo indígena, and not “very wealthy”
- **Net income over the last month in 2017.** Participants with higher net incomes in 2017 preferred candidate friends not from the same valley and not from La Paz, but from the same pueblo indígena, and not “very wealthy”
- **Net income over the last month in 2014-15.** Participants with higher net incomes in 2014–15 preferred candidate friends not from the same valley and not from La Paz, and not “very wealthy”
- **Difference in net income between 2017 and 2014-15.** Participants with a larger discrepancy in their net income between 2017 and 2014–15 preferred candidate friends not from the same valley and not from La Paz, from the same pueblo indígena, and not “very wealthy"

- **Non-anonymous game play**. Participants who played the game non-anonymously (that is, sharing their name and group affiliation with the recipient) preferred candidate friends not from the same valley and not from La Paz, from the same pueblo indígena, and not "very wealthy"

The four-way interactions between controls and the predictors of interest (described as Models 1 and 2 in Section 1.4) are complicated to interpret, so we refrain from doing so here and refer interested readers to the model code (available at www.github.com/annethro/parochialism).

References

Hideo Aizaki. Basic functions for supporting an implementation of choice experiments in R. *Journal Of Statistical Software*, 50(2):1–24, 2012. URL http://www.jstatsoft.org/v50/c02/.

Paul Christian Bürkner. brms: An R package for Bayesian multilevel models using Stan. *Journal of Statistical Software*, 80(1), 2017. ISSN 15487660. .

Richard McElreath. *Statistical rethinking*. CRC press, 2016.

Anne C Pisor and Michael Gurven. Risk buffering and resource access shape valuation of out-group strangers. *Scientific reports*, 6(1):1–10, 2016.

Anne C Pisor and Michael Gurven. When to diversify, and with whom? *Evolution and Human Behavior*, 39(1):30–39, 2018.

Anne C. Pisor and James Holland Jones. Do people manage climate risk through long-distance relationships? *American Journal of Human Biology*, (e23525):e23525, 2020. . URL https://doi.org/10.1002/ajhb.23525.

R Core Team. *R: A Language and Environment for Statistical Computing*. R Foundation for Statistical Computing, Vienna, Austria, 2016. URL https://www.R-project.org/.

Vithala R Rao et al. *Applied conjoint analysis*. Springer, 2014.

Stan Development Team. RStan, 2019. URL http://mc-stan.org/. Version 2.19.2.

Stef Van Buuren and Karin Groothuis-Oudshoorn. Multivariate imputation by chained equations. *Journal Of Statistical Software*, 45(3): 1–67, 2011. ISSN 15487660. . URL http://igitur-archive.library.uu.nl/fss/2010-0608-200146/UUindex.html.

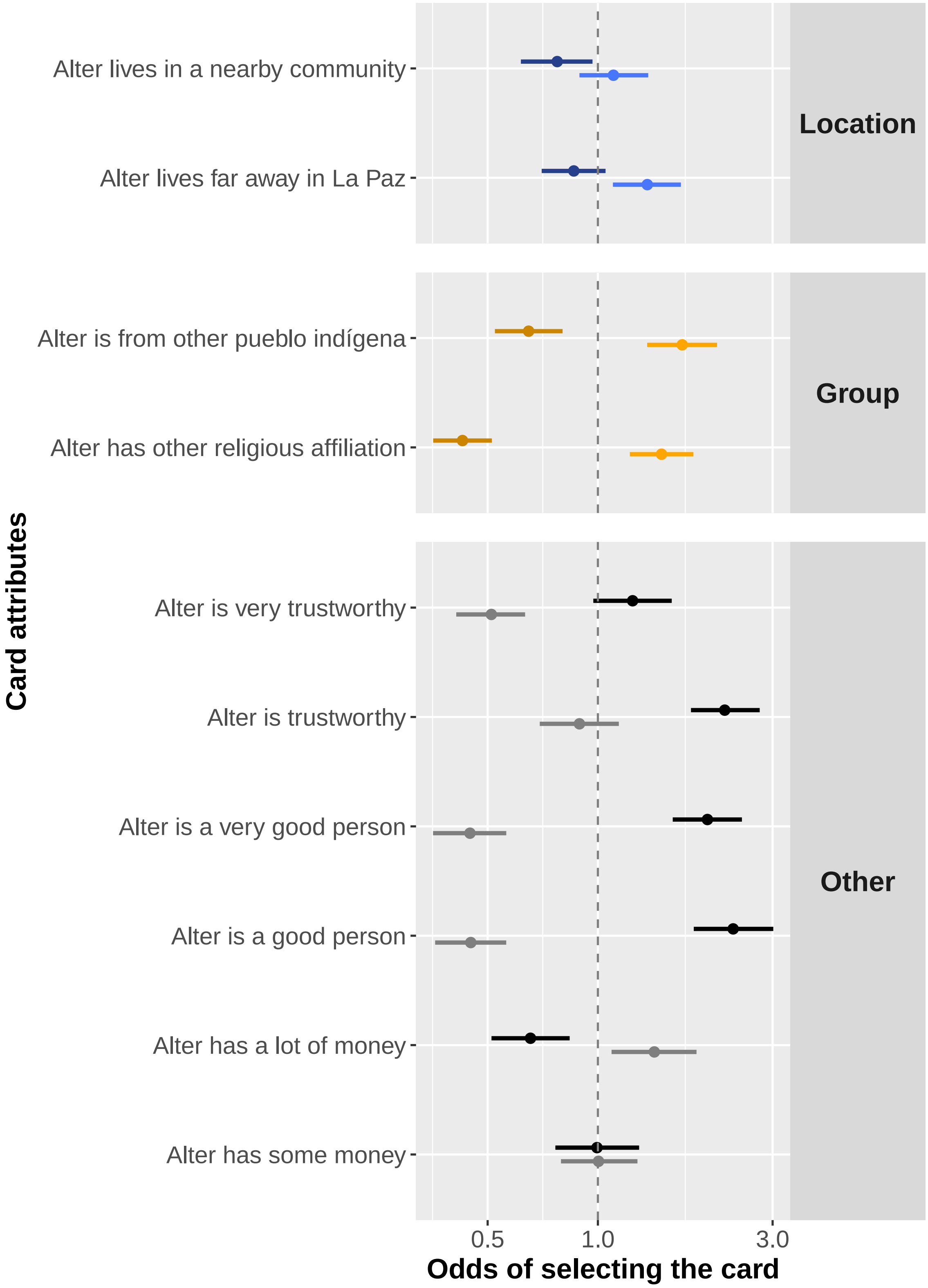

Fig. S2: Each participant was presented with a pair of hypothetical individuals and was asked which they preferred as a new friend. Each participant evaluated 18 pairs of individuals. Here, we present non-standardized estimates (means and 90% credible regions) from a Bernoulli regression; these are the odds of selecting one individual if it were to differ by only a single attribute from the other individual. Different from the main text, we provide estimates for both picking the left-hand card (light colors) and the right-hand card (dark colors). The base case is a candidate friend who lives in the same community, is from the same pueblo indígena and has the same religious affiliation, and who is not good, not trustworthy, and has no money.

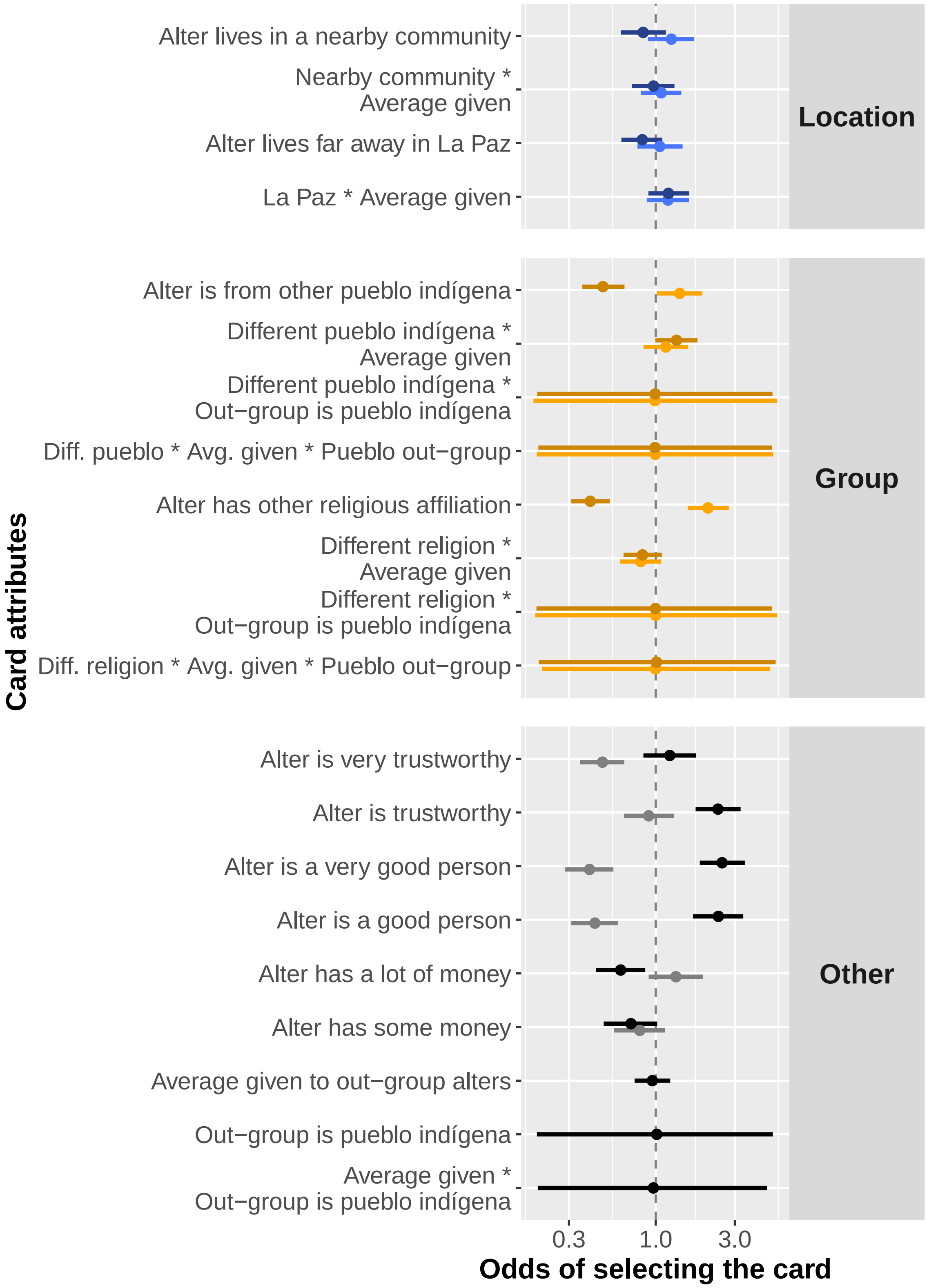

Fig. S3: A model including interactions between (i) average amount given to out-group members in 2014–15, (ii) whether those out-group members were from a religious out-group or pueblo indígena out-group, and (iii) from the card choice task: where the candidate friend lived, their religious affiliation, and pueblo indígena; we include a three-way interaction between (i), (ii), and (iii) for a candidate friend's religious affiliation and pueblo indígena, but only a two-way interaction between (i) and (iii) for location, as participants were not told recipients' locations in the 2014–15 game. Estimates are non-standardized (means and 90% credible regions) from a logistic regression. We include estimates for both picking the left-hand card (light colors) and the right-hand card (dark colors).
